# Осложнения псевдогипопаратиреоза и врожденных форм гипопаратиреоза у детей

**DOI:** 10.14341/probl13704

**Published:** 2026-05-20

**Authors:** А. А. Джамалудинова, Л. С. Созаева, Н. В. Маказан, А. А. Колодкина, К. С. Куликова, Н. Ю. Калинченко, А. В. Болмасова, Е. Э. Новокрещенных, В. А. Петеркова, Н. Г. Мокрышева

**Affiliations:** Национальный медицинский исследовательский центр эндокринологии им. академика И.И. Дедова; Endocrinology Research Centre; Национальный медицинский исследовательский центр эндокринологии им. академика И.И. Дедова; Медико-генетический научный центр им. академика Н.П. Бочкова; РДКБ — филиал ФГАОУ ВО РНИМУ им. Н.И. Пирогова; Endocrinology Research Centre; Research Center for Medical Genetics; Russian Children's Clinical Hospital, Pirogov RNIMU

**Keywords:** гипопаратиреоз, дети, врожденные формы, псевдогипопаратиреоз, осложнения, hypoparathyroidism, children, congenital forms, pseudohypoparathyroidism, complications

## Abstract

**ОБОСНОВАНИЕ:**

ОБОСНОВАНИЕ. Гипопаратиреоз (ГПТ) и псевдогипопаратиреоз (ПГПТ) — редкие, преимущественно генетически обусловленные заболевания у детей, проявляющиеся гипокальциемией и гиперфосфатемией. ГПТ и ПГПТ имеют схожие осложнения: кальцификация головного мозга и хрусталика. При ГПТ также наблюдается повышенный риск развития нефрокальциноза. Данные о частоте и структуре осложнений у детей в России ограничены, а сравнительные исследования отсутствуют.

**ЦЕЛЬ:**

ЦЕЛЬ. Сравнить частоту хронических осложнений и определить факторы, ассоциированные с их развитием, у детей с ПГПТ и врожденными формами ГПТ.

**МАТЕРИАЛЫ И МЕТОДЫ:**

МАТЕРИАЛЫ И МЕТОДЫ. Ретроспективное исследование с проспективным компонентом включало 135 детей с ПГПТ и врожденными формами ГПТ. Оценены результаты лабораторно-инструментальных исследований.

**РЕЗУЛЬТАТЫ:**

РЕЗУЛЬТАТЫ. У 82% детей выявлено хотя бы одно осложнение, ассоциированное с гипопаратиреозом (ГПТ) и псевдогипопаратиреозом (ПГПТ). Нефрокальциноз обнаруживался наиболее часто у пациентов с аутоиммунным полигландулярным синдромом (АПС) 1-го типа (67%) и с аутосомно-доминантной гипокальциемией (АДГ) 1-го типа (62%) и гораздо реже у пациентов с ПГПТ (22%) и неуточненными формами ГПТ (18%). Продолжительность заболевания и длительность терапии активными аналогами витамина D и препаратами кальция была достоверно дольше у пациентов с нефрокальцинозом (p<0,001). Достоверной связи между гиперкальциурией и развитием нефрокальциноза не получено (р=0,567). У 48,9% пациентов наблюдалось снижение рСКФ, соответствующее хронической болезни почек (ХБП) 2 стадии. Синдром Фара наблюдался в 76% случаев, преимущественно с поражением базальных ганглиев, а наличие минеральных отложений ассоциировалось с гиперфосфатемией (p=0,010). Катаракта и микронефролитиаз встречались реже (18,6% и 3,9% соответственно), без значимых различий между нозологическими формами. Чаще наблюдались помутнения кортикального (68%) и заднесубкапсулярного (41%) слоев хрусталика. Длительность заболевания была выше в группе с катарактой (p=0,018).

**ЗАКЛЮЧЕНИЕ:**

ЗАКЛЮЧЕНИЕ. Среди осложнений у детей наиболее часто регистрировались нефрокальциноз, гиперкальциурия и синдром Фара. Почечные осложнения преимущественно встречались у пациентов с АПС 1-го типа и АДГ 1-го типа. Гиперкальциурия сохранялась даже при оптимальных уровнях сывороточного кальция у большинства пациентов.

## Abstract (en)

**BACKGROUND:**

BACKGROUND: Hypoparathyroidism (HypoPT) and pseudohypoparathyroidism (PHP) are rare, predominantly genetically determined diseases in children with similar complications, characterized by hypocalcemia and hyperphosphatemia. PHP and HypoPT have similar complications: brain and lens calcification. In HypoPT, there is also an increased risk of developing nephrocalcinosis. Data on the frequency and structure of complications in affected children in Russia are limited, and comparative studies are lacking.

**AIM:**

AIM: To compare the prevalence of chronic complications and to identify factors associated with their development in children with PHP and congenital forms of HypoPT.

**MATERIALS AND METHODS:**

MATERIALS AND METHODS: A retrospective study with a prospective component included 135 children with PHP and congenital forms of HypoPT. The results of laboratory and instrumental studies were evaluated.

**RESULTS:**

RESULTS: At least one complication associated with HypoPT/PHP was identified in 82% of children. Nephrocalcinosis was most frequently detected in patients with autoimmune polyendocrine syndrome type 1 (APS-1) (67%) and autosomal dominant hypocalcemia type 1 (ADH1), and much less often in patients with PHP (22%) and unspecified forms of HypoPT (18%). Disease duration, as well as the duration of therapy with active vitamin D analogs and calcium supplements, was significantly longer in patients with nephrocalcinosis (p < 0.001). An association between nephrocalcinosis and cataracts was established (p=0.005). Hypercalciuria persisted in 60% of patients despite medical compensation, was most prevalent in APS-1 and ADH1, and was not observed in PHP (p < 0.001). No statistically significant relationship was identified between hypercalciuria and the development of nephrocalcinosis (p=0.567). A decrease in eGFR corresponding to CKD stage 2 was observed in 48.9% of patients. Fahr’s syndrome was detected in 76% of cases, predominantly involving the basal ganglia, and the presence of mineral deposits was associated with hyperphosphatemia (p=0.010). Cataract and micronephrolithiasis were less frequent (18.6% and 3.9%, respectively), with no significant differences between nosological groups. The most common lens opacities observed were cortical (68%) and posterior subcapsular (41%). The duration of the disease was longer in patients with cataracts (p = 0.018).

**CONCLUSION:**

CONCLUSION: Among the complications observed in children, nephrocalcinosis, hypercalciuria, and Fahr’s syndrome were the most frequent. Renal complications predominated in patients with APS-1 and ADH1. Hypercalciuria persisted despite optimal serum calcium levels in most patients.

## ОБОСНОВАНИЕ

Гипопаратиреоз (ГПТ) — группа заболеваний, характеризующихся гипокальциемией вследствие снижения секреции паратиреоидного гормона (ПТГ). Наиболее распространенной является приобретенная форма ГПТ как следствие удаления или повреждения околощитовидных желез при тиреоидэктомии, и преимущественно наблюдается у взрослых пациентов [1]. У детей же чаще имеют место врожденные формы ГПТ, в основе которых — генетические причины [2]. Распространенность нехирургических форм гипопаратиреоза по данным различных исследований составляет 1,8–2,3 случая на 100 000 населения [3][4].

Псевдогипопаратиреоз (ПГПТ) — это наследственное заболевание с мультигормональной резистентностью и особенностями фенотипа. Распространенность ПГПТ оценивается как 1,1–1,2 случая на 100 000 населения [3][5].

ГПТ и ПГПТ имеют общие биохимические признаки — гипокальциемию и гиперфосфатемию — и ряд сходных осложнений, включая кальцификацию структур головного мозга и катаракту [4–6]. При ГПТ также повышен риск формирования нефрокальциноза, нефролитиаза и прогрессирования хронической болезни почек, что не характерно для ПГПТ [5][7]. Гиперкальциурия, возникающая на фоне приема препаратов кальция и активных аналогов витамина D, является дополнительным фактором риска почечных осложнений при ГПТ. Развитие осложнений связывают как с длительной гиперфосфатемией при недиагностированном и/или некомпенсированном ГПТ и ПГПТ, так и с проводимой терапией, преимущественно у пациентов с ГПТ [8][9].

В России осложнения ГПТ и ПГПТ у детей малоизучены. Опубликованы работы, описывающие спектр осложнений у детей с ПГПТ и у детей с одной из причин ГПТ — АПС 1‑го типа [10][11]. Нет исследований, которые объединяли бы анализ осложнений ПГПТ и врожденных форм ГПТ, а также не проводилось их сравнительное изучение. При этом данные такого исследования позволят углубить понимание клинического течения этой группы заболеваний и могут способствовать оптимизации лечения и наблюдения за этими пациентами.

## ЦЕЛЬ ИССЛЕДОВАНИЯ

Оценить и сравнить структуру и частоту хронических осложнений и определить факторы, ассоциированные с их развитием, у детей с гипокальциемией вследствие ПГПТ и врожденных форм ГПТ.

## МАТЕРИАЛЫ И МЕТОДЫ

## Место и время проведения исследования

Место проведения. Институт детской эндокринологии ГНЦ РФ ФГБУ «НМИЦ эндокринологии им. академика И.И. Дедова» Минздрава России.

Время исследования. сбор данных для исследования проводился в период с сентября 2015-го по июль 2025-го гг.

## Изучаемые популяции

Популяция: в исследование включено 135 пациентов (61 девочка, 74 мальчика) в возрасте от 2 дней до 18 лет на момент постановки диагноза. В ходе исследования пациентам проводилось обследование в ФГБУ «НМИЦ эндокринологии им. академика И.И. Дедова» Минздрава России и/или оценивались их медицинские документы с исследованиями, проведенными в других клиниках.

Критерии включения: пациенты с подтвержденной хронической гипокальциемией вследствие псевдогипопаратиреоза и врожденных форм гипопаратиреоза.

Критерии исключения: пациенты с хронической почечной недостаточностью, приобретенными формами гипокальциемии вследствие медицинских вмешательств (тиреоидэктомия или облучение области шеи).

## Способ формирования выборки из изучаемой популяции

Сплошной способ формирования выборки.

## Дизайн исследования

Одноцентровое одновыборочное ретроспективное сравнительное исследование с проспективным компонентом.

## Методы

Протокол исследования включал в себя оценку жалоб, сбор анамнеза жизни и заболевания, наследственного анамнеза, оценку антропометрических данных, анализ медицинских документов пациента.

Учитывая то, что нередко гипокальциемия устанавливается позднее, после появления первых клинических симптомов, а осложнения могут быть связаны с длительностью заболевания, то при расчете возраста манифестации заболевания и длительности заболевания проводился анализ от даты первого лабораторного подтверждения гипокальциемии и от даты предполагаемого начала заболевания (первый судорожный синдром без установления гипокальциемии): у некоторых пациентов эти даты совпадали, а у некоторых разница составляла годы.

Для оценки частоты осложнений в зависимости от причины заболевания пациенты были разделены на 5 нозологических групп: АПС 1‑го типа, ПГПТ, АДГ 1‑го типа, ГПТ неясного генеза и другие наследственные формы ГПТ. В последнюю группу вследствие ограниченного числа случаев объединены редкие врожденные варианты ГПТ — синдром Бараката, синдром делеции 22q11.2 и синдром Кенни-Каффи.

Лабораторная диагностика проводилась в диагностической лаборатории ФГБУ «НМИЦ эндокринологии им. академика И.И. Дедова» Минздрава России. Часть данных (биохимического анализа крови) получена ретроспективно из медицинских документов пациентов, предоставленных при амбулаторном приеме или в рамках телемедицинского консультирования.

Оценивались уровни ПТГ (норма 15–65 пг/мл), общего кальция (дети до 2-х лет — 2,25–2,75 ммоль/л, 2–12 лет — 2,20–2,70 ммоль/л, 13–18 лет — 2,10–2,55 ммоль/л), ионизированного кальция (1,03–1,29 ммоль/л), неорганического фосфата [12], креатинина (дети до года — 18–35 мкмоль/л, 1–14 лет — 27–62 мкмоль/л, 14–18 лет — 44–88 мкмоль/л) в сыворотке крови. Соотношение кальций/креатинин рассчитывалось по показателю их содержания в разовой порции мочи (0–6 мес. 0,1–2,60; 6–12 мес. — 0,09–2,20; 1–2 года — 0,07–1,50; 2–3 года — 0,06–1,40; 3–5 лет — 0,05–1,10; 5–7 лет — 0,04–0,80; 5–7 лет — 0,04–0,80; 7–10 лет — 0,04–0,70; 10–17 лет — 0,04–0,60 ммоль/ммоль). Биохимический анализ выполнялся на автоматическом анализаторе Architect c 8000 (Abbott, США). Фильтрационная функция почек оценивалась на основании расчета скорости клубочковой фильтрации (рСКФ) по формуле Шварца [13]; стадия хронической болезни почек (ХБП) устанавливалась в соответствии с классификацией KDIGO (Kidney Disease: Improving Global Outcomes) [14]. Критерием гипокальциемии считалось снижение уровня общего и/или ионизированного кальция крови ниже референсных значений.

При оценке экскреции кальция с мочой на фоне терапии у пациентов с ГПТ/ПГПТ оптимальным значением кальция сыворотки взят уровень ниже середины (≤1,16 ммоль/л) и несколько ниже нижней границы стандартных референсных значений (≤1,00 ммоль/л). Не учитывались биохимические результаты мочи пациентов, получавших препараты, влияющие на экскрецию кальция и кислотно-щелочной баланс (гидрохлоротиазид, цитратсодержащие средства), из-за риска ложноотрицательных результатов. Показатели соотношения кальций/креатинин (Са/Cr) мочи, превышающие возрастные нормы при оптимальном уровне кальция сыворотки крови, расценивались как гиперкальциурия. На первом этапе из анализа исключены пациенты с АДГ 1‑го типа и ПГПТ по патогенетическим причинам, обусловленным высоким и, напротив, низким риском гиперкальциурии в данных группах.

На втором этапе, учитывая различия в патогенезе, мы проанализировали экскрецию кальция на фоне терапии между группами пациентов с высоким — АДГ 1‑го типа и низким риском ее развития — ПГПТ, и с остальными формами ГПТ (АПС 1‑го типа, с неуточненной причиной и другими синдромами), у которых экскреция кальция в основном зависит от заместительной терапии. Это позволило оценить вклад патогенетических механизмов в развитие гиперкальциурии или ее отсутствие независимо от проводимой терапии.

Для изучения связи синдрома Фара с гиперфосфатемией оценивались уровень неорганических фосфатов в крови на момент диагностики синдрома Фара, предполагаемая длительность заболевания и длительность заболевания с момента впервые задокументированной гипокальциемии.

Инструментальные исследования проводились как в условиях ФГБУ «НМИЦ эндокринологии им. академика И.И. Дедова» Минздрава России, так и в других лечебных учреждениях и включали: ультразвуковое исследование (УЗИ) почек, мультиспиральную компьютерную томографию (МСКТ) и магнитно-резонансную томографию (МРТ) головного мозга. Критерием нефрокальциноза считалось обнаружение диффузно повышенной эхогенности паренхимы почек. Микрокальцинаты диагностировались при визуализации гиперэхогенных включений с акустической тенью диаметром до 4 мм.

Офтальмологическое обследование включало стандартную проверку остроты зрения, осмотр переднего отрезка глаза с использованием щелевой лампы и офтальмоскопию глазного дна.

## Статистический анализ

Расчет данных производился с помощью статистического пакета Statistica 13 (StatSoft inc., США), MS Exel 2016 (Microsoft, США). Количественные результаты представлены в виде медианы (Ме) и квартилей [ Q1; Q3], соответствующие 25 и 75 перцентилям. Качественные данные представлены в виде абсолютных (n) и относительных (%) частот. Для оценки статистически значимых различий между независимыми группами по количественным признакам использованы критерии Манна-Уитни (U-test) и Краскела-Уоллиса, выбранный критерий приведен в комментариях к таблицам с результатами. Для выявления статистически значимых различий для качественных признаков использован двухсторонний точный критерий Фишера (ТКФ2), при анализе таблиц сопряженности больше 2х2 — критерий Фишера-Фримана-Холтoна. Критический уровень значимости различий принимали р<0,05. При множественных сравнениях применяли поправку Бонферрони (P0), после чего значения р в диапазоне между рассчитанным р0 и 0,05 интерпретировались как статистическая тенденция.

## Этическая экспертиза

Исследование одобрено Локальным этическим комитетом ФГБУ «НМИЦ эндокринологии им. академика И.И. Дедова» Минздрава России (Протокол №16 от 13.09.2023 г.).

## РЕЗУЛЬТАТЫ

Среди 135 включенных в исследование пациентов с гипокальциемией наиболее частыми причинами ее развития являлись: аутоиммунный полигландулярный синдром (АПС) 1‑го типа — 42,2% (57/135), псевдогипопаратиреоз — 25,9% (35/135) и аутосомно-доминантная гипокальциемия (АДГ) 1‑го типа — 10,4% (14/135). Редкие генетические синдромы (синдром Бараката, синдром делеции 22q11.2, синдром Кенни-Каффи) составили 13,4% (18/135), а в 8,1% (11/135) случаев причину ГПТ установить не удалось.

Возраст предполагаемого начала заболевания составил 4,5 года [ 2,5; 9,4]. Гипокальциемия впервые регистрировалась в 6,1 года [ 3,2; 10,8]. Предполагаемая длительность заболевания по клиническим данным составила 4,7 года [ 2,0; 8,3], а длительность заболевания по впервые подтвержденной гипокальциемии - 3,0 года [ 1,3; 7,2].

Хотя бы одно осложнение имели 82% (111/135) пациентов.

Гиперкальциурия наблюдалась преимущественно у больных с АПС 1‑го типа (75%) и АДГ 1‑го типа (50%) и отсутствовала при ПГПТ (0%) (p<0,001). Наибольшая распространенность нефрокальциноза отмечена у пациентов с АПС 1‑го типа (67%) и АДГ 1‑го типа (64%), в то время как в группе с ПГПТ это осложнение диагностировано реже (22%), а при ГПТ неясного генеза — лишь в 18% наблюдений (p<0,001). По остальным осложнениям, включая синдром Фара (p=0,816), нефролитиаз (p=0,097), снижение рСКФ (<90 мл/мин/1,73 м²) (p=0,302) и катаракту (p=0,028), статистически значимых различий между нозологическими группами не получено (p>0,005 после поправки Бонферрони) (табл. 1).

**Table:** Таблица 1. Частота осложнений у пациентов в зависимости от нозологической формы заболевания (N=135)* Применена поправка Бонферрони: Р0=0,05/10=0,005 *Различное количество пациентов по отдельным осложнениям связано с тем, что исследование включало как ретроспективные данные, так и данные медицинских документов пациентов с проведенными исследованиями по месту жительства, в которых не все исследования были проведены или указаны. **Группа “другие наследственные формы ГПТ” включала синдром Бараката, синдром делеции 22q11.2 и синдром Кенни-Каффи.

Осложнения ГПТ	Нозологическая форма гипокальциемии	Р,Критерий Фишера-Фримана-Холтона
АПС 1‑го типа(1)	ПГПТ(2)	АДГ 1‑го типа(3)	Другие наследственные формы гипопаратиреоза(4)**	Гипопаратиреоз неясного генеза(5)
	Наличие признака	
Нефрокальциноз двусторонний	37 (67%)N=55	7 (22%)N=32	9 (64%)N=14	7 (47%)N=15	2 (18%)N=11	<0,001p1-2<0,001p1-3=1,000p1-4=0,227p1-5=0,005p2-3=0,008p2-4=0,100p2-5=1,000p3-4=0,462p3-5=0,419p4-5=0,217
Нефролитиаз	2 (4%)N=55	0 (0%)N=32	0 (0%)N=14	1 (7%)N=15	2 (18%)N=11	0,097
Гиперкальциурия	27 (75%)N=36	0 (0%)N=15	4 (50%)N=8	1 (14%)N=7	2 (40%)N=5	<0,001p1-2<0,001p1-3=0,209p1-4=0,005p1-5=0,139p2-3=0,008p2-4=0,318p2-5=0,053p3-4=0,282p3-5=1,000p4-5=0,523
Синдром Фара	21 (72%)N=29	14 (70%)N=20	7 (88%)N=8	7 (78%)N=9	4 (100%)N=4	0,816
Катаракта	17 (30%)N=56	3 (9%)N=32	3 (21%)N=14	1 (6%)N=17	0 (0%)N=10	0,028
рСКФ <90 мл/мин/1,73 м² по формуле Шварца	30 (53%)N=57	13 (38%)N=34	5 (36%)N=14	9 (56%)N=16	7 (70%)N=10	0,302

## Оценка экскреции кальция с мочой

На первом этапе исследования у 60% пациентов (30/50) выявлена гиперкальциурия на фоне оптимальных значений кальция крови с учетом возрастных норм.

Терапия активными аналогами витамина D (альфакальцидол или кальцитриол) проводилась всем пациентам, у одного пациента применялись оба препарата одновременно. Статистически значимых различий между группами с гиперкальциурией и без не выявлено (p>0,006 после поправки Бонферрони) (табл. 2). Но отмечалась тенденция к более высоким суточным дозам альфакальцидола у пациентов с гиперкальциурией: 2 мкг против 1,25 мкг у пациентов без гиперкальциурии (p=0,037 до введения поправки Бонферрони).

**Table:** Таблица 2. Сравнительный анализ признаков пациентов с наличием и отсутствием гиперкальциурии Примечание. В анализ не включены пациенты с АДГ 1‑го типа и ПГПТ.*Из статистического анализа исключен один пациент, получавший одновременно альфакальцидол и кальцитриол.Применена поправка Бонферрони: Р0=0,05/9=0,006¹ U-test² Критерий Фишера-Фримана-Холтона

Признак	Группа с гиперкальциурией	Группа без гиперкальциурии	p
N	Me [ Q1; Q3]	N	Me [ Q1; Q3]
Возраст пациента, лет	30	11 [ 9; 14]	20	7 [ 5; 13]	0,118¹
Креатинин, мкмоль/л	30	58,35 [ 51,30; 62,90]	18	49,45 [ 44; 53,4]	0,059¹
рСКФ по Шварцу, мл/мин/1,73 м²	30	86 [ 82; 95]	18	90 [ 79; 93]	0,766¹
Длительность терапии активными аналогами витамина D, лет	30	4,2 [ 1,0; 7,0]	18	2,5 [ 1,7; 4,0]	0,139¹
Длительность терапии препаратами кальция, лет	20	4,2 [ 1,4; 7,2]	11	2,2 [ 0,8; 5,1]	0,397¹
Альфакальцидол, мкг/сут	23*	2,00 [ 1,50; 3,50]	18	1,25 [ 1,00; 2,25]	0,037¹
Альфакальцидол, мкг/кг/сут	23*	0,055 [ 0,043; 0,075]	18	0,051 [ 0,028; 0,083]	0,486¹
Кальцитриол, мкг/сут	6*	1,5 [ 1,0; 1,5]	0	-	
Кальцитриол, мкг/кг/сут	6*	0,049 [ 0,027; 0,077]	0	-	
Препараты кальция, мг/сут	20	1000 [ 750; 1500]	12	1500 [ 1000; 2000]	0,161¹
Препараты кальция, мг/кг/сут	20	32,6 [ 18,9; 44,4]	12	45,2 [ 24,3; 102,4]	0,150¹
	N	n (%)	N	n (%)	
Стадия ХБП- Стадия 1- Стадия 2- Стадия 3а	30	12 (40%)17 (56,7%)1 (3,3%)	18	9 (50%)9 (50%)	0,852²

На втором этапе исследования высокая распространенность гиперкальциурии обнаружена при АДГ 1‑го типа (50%) и при других нехирургических формах ГПТ (62,5%) и ее отсутствие при ПГПТ (0%) (p<0,001) (табл. 3). Надо отметить, что в представленной группе нехирургических форм гиперкальциурия чаще всего встречалась у пациентов с АПС 1‑го типа. Таким образом, гиперкальциурия при оптимальных значениях кальция более всего характерна для пациентов с АПС 1‑го типа и АДГ 1‑го типа.

**Table:** Таблица 3. Сравнительная характеристика пациентов с АДГ 1‑го типа, ПГПТ и остальными формами нехирургического ГПТ по гиперкальциурии. ¹ Критерий Краскела-Уоллиса² Критерий Фишера-Фримана-Холтона

Признак	Другие формы нехирургического ГПТ (N=48)(1)	АДГ 1‑го типа (N=8)(2)	ПГПТ(N=15)(3)	p
Соотношение кальций/креатинин разовой порции мочи, ммоль/ммоль(Me [ Q1; Q3])	0,90 [ 0,54; 1,18]	0,81 [ 0,50; 1,39]	0,07 [ 0,06; 0,12]	<0,001¹p1-2=1,000p1-3<0,001p2-3<0,001
Гиперкальциурия (n (%))	30 (62,5%)	4 (50%)	0 (0%)	<0,001²

## Нефрокальциноз и мочекаменная болезнь (МКБ)

По данным УЗИ почек, нефрокальциноз выявлен у 48,8% (62/127), микронефролитиаз — у 3,9% (5/127) пациентов. Во всех случаях нефрокальциноз имел двустороннюю локализацию. Микронефролитиаз был двусторонним в трех наблюдениях и односторонним — в двух. У трех пациентов нефрокальциноз сочетался с микронефролитиазом.

В группе пациентов с нефрокальцинозом гиперкальциурия выявлена у 64% (18/28) пациентов и у 55% (12/22) без него. Статистически значимых различий между пациентами с нефрокальцинозом и без него по гиперкальциурии не получено (p=0,567) (табл. 4).

**Table:** Таблица 4. Взаимосвязь нефрокальциноза с другими осложнениями ГПТ/ПГПТ Применена поправка Бонферрони: Р0=0,05/3=0,017

Признак	Группа с нефрокальцинозом	Группа без нефрокальциноза	p, ТКФ2
N	n (%)	N	n (%)	
Гиперкальциурия	28	18 (64%)	22	12 (55%)	0,567
Синдром Фара	36	28 (78%)	32	23 (72%)	0,589
Катаракта	62	18 (29%)	62	5 (8%)	0,005

Учитывая предположительно схожие патогенетические механизмы формирования эктопической кальцификации, проведен поиск возможной связи между нефрокальцинозом и другими осложнениями. Выявлена статистически значимая ассоциация с катарактой, которая достоверно чаще встречалась у пациентов с нефрокальцинозом (p=0,005) (табл. 4).

При сравнительном анализе пациентов с нефрокальцинозом и без него выявлено, что продолжительность заболевания и длительность терапии активными аналогами витамина D и препаратами кальция была достоверно выше в группе с нефрокальцинозом (p<0,001) (табл. 5). Частота выявления нефрокальциноза в зависимости от нозологической формы гипокальциемии представлена в таблице 1.

**Table:** Таблица 5. Сравнительный анализ признаков пациентов с наличием и отсутствием нефрокальциноза Применена поправка Бонферрони: P0=0,05/6=0,008¹ U-test² Критерий Фишера-Фримана-Холтона

Признак	Группа с нефрокальцинозом	Группа без нефрокальциноза	p
N	Me [ Q1; Q3]	N	Me [ Q1; Q3]
Креатинин, мкмоль/л	62	57,2 [ 49,0; 8,2]	62	55,1 [ 49,9; 6,3]	0,778¹
рСКФ по Шварцу, мл/мин/1,73 м²	62	88 [ 79; 95]	62	91 [ 83; 100]	0,118¹
Длительность заболевания с момента впервые задокументированной гипокальциемии, лет	62	5,8 [ 2,4; 8,2]	65	2,2 [ 0,6; 5,2]	<0,001¹
Предполагаемая длительность заболевания, лет	62	6,5 [ 3,7; 9,9]	65	3,2 [ 1,2; 6,7]	<0,001¹
Длительность терапии активными аналогами витамина D, лет	62	5,0 [ 2,0; 7,8]	64	1,5 [ 0,3; 4,3]	<0,001¹
Длительность терапии препаратами кальция, лет	55	5,1 [ 2,1; 8,0]	54	1,8 [ 0,4; 4,8]	<0,001¹
	N	n (%)	N	n (%)	
Стадия ХБП- Стадия 1- Стадия 2- Стадия 3а- Стадия 4	62	27 (44%)32 (52%)2 (3%)1 (1%)	62	36 (58%)25 (40%)0 (0%)1 (2%)	0,232²

У 48,9% (64/131) пациентов наблюдалось снижение рСКФ <90 мл/мин/1,73 м², соответствующее ХБП 2 стадии. Группы пациентов с оптимальным значением рСКФ (≥90 мл/мин/1,73 м²) и со сниженной рСКФ (<90 мл/мин/1,73 м²) статистически не различались (табл. 6). Анализ показателей рСКФ не выявил различий между нозологическими формами гипокальциемии (табл. 1).

**Table:** Таблица 6. Сравнительный анализ показателей пациентов с оптимальным значением рСКФ (≥90 мл/мин/1,73 м²) и снижением рСКФ<90 мл/мин/1,73 м² Применена поправка Бонферрони: P0=0,05/4=0,013

Признак	Группа с рСКФ<90 мл/мин/1,73 м²	Группа с рСКФ≥90 мл/мин/1,73 м²	P, U-test
N	Me [ Q1; Q3]	N	Me [ Q1; Q3]
Длительность заболевания с момента впервые задокументированной гипокальциемии, лет	64	3,2 [ 1,5; 7,9]	67	3,9 [ 1,2; 7,5]	0,809
Предполагаемая длительность заболевания, лет	64	5,1 [ 1,7; 9,9]	67	4,9 [ 2,2; 7,8]	0,991
Длительность терапии активными аналогами витамина D, лет	64	2,8 [ 1,2; 6,5]	67	2,4 [ 0,6; 6,6]	0,438
Длительность терапии препаратами кальция, лет	56	3,6 [ 1,3; 7,9]	56	3,2 [ 0,9; 5,7]	0,367

## Синдром Фара

Синдром Фара был диагностирован у 76% (53/70) детей на основании данных мультиспиральной компьютерной томографии (МСКТ) или магнитно-резонансной томографии (МРТ) головного мозга. У 24% (17/70) пациентов патологических изменений структур головного мозга выявлено не было. Наиболее часто кальцификации были локализованы в базальных ганглиях (42,1%), затем — в лобных долях (25,4%), теменных долях (12,3%) и мозжечке (11,4%). Поражение таламуса и височных долей встречалось значительно реже. У 5 пациентов, чьи данные оценивались по медицинским документам, данные о локализации отсутствовали, а у 10 пациентов — отложения описывали в субкортикальных слоях головного мозга без уточнения анатомического расположения.

Частота синдрома Фара не зависела от нозологической формы гипокальциемии (табл. 1).

Статистически достоверных различий между группами с синдромом Фара и без по уровню неорганических фосфатов не получено (p=0,021) (p>0,013 после поправки Бонферрони) (табл. 7). Данный способ имел значимое ограничение, связанное с тем, что уровень фосфатов зависит от текущей степени компенсации заболевания и является вариабельным показателем.

**Table:** Таблица 7. Сравнительная характеристика признаков пациентов с наличием и отсутствием синдрома Фара Применена поправка Бонферрони: P0=0,05/4=0,013

Признак	Группа с синдромом Фара	Группа без синдрома Фара	P, U-test
N	Me [ Q1; Q3]	N	Me [ Q1; Q3]
Неорганические фосфаты сыворотки крови, ммоль/л	51	2,66 [ 2,00; 3,27]	16	2,00 [ 1,72; 2,48]	0,021
Фосфаты сыворотки крови/верхняя граница нормы (по возрасту и полу)	51	1,39 [ 1,05; 1,67]	16	1,05 [ 0,94; 1,23]	0,010
Длительность заболевания с момента впервые задокументированной гипокальциемии, лет	53	4,8 [ 1,6; 7,9]	17	6,6 [ 1,3; 8,2]	0,880
Предполагаемая длительность заболевания, лет	53	6,5 [ 3,9; 11,2]	17	6,7 [ 2,3; 8,4]	0,502

Поскольку у детей референсный уровень фосфатов в крови варьирует в зависимости от возраста и пола, для корректного сравнения групп показатели фосфатов были унифицированы как отношение фактического уровня фосфатов к верхней границе возрастно-половой нормы. По полученному соотношению выявлены статистически значимые различия, у пациентов с синдромом Фара отношение уровня фосфатов к норме оказалось выше: 1,39 [ 1,05; 1,67] против 1,05 [ 0,94; 1,23] (p=0,010) (табл. 7).

Группы с синдромом Фара и без также статистически не различались по длительности заболевания с момента впервые задокументированной гипокальциемии и предполагаемой длительности заболевания (p>0,013 после поправки Бонферрони).

В группе пациентов с синдромом Фара катаракта наблюдалась в 25% (13/52) случаев и в 6% (1/16) в группе без него, статистически достоверной связи синдрома Фара и катаракты не получено (p=0,161, двусторонний точный критерий Фишера). Кроме того, синдром Фара не был ассоциирован с нефрокальцинозом (табл. 4).

## Катаракта

По результатам офтальмологического осмотра катаракта диагностирована у 18,6% (24/129). Среди пациентов с катарактой у 29,2% (7/24) она была уже к моменту манифестации заболевания до начала терапии. Полные данные офтальмологического обследования были доступны у 22 из 24 пациентов с катарактой. У 8 пациентов было поражение более одного слоя хрусталика. Наиболее часто регистрировались помутнения кортикального слоя (68%, 15/22), далее — заднесубкапсулярные изменения (41%, 9/22), переднесубкапсулярные (27%, 6/22) и ядерные (23%, 5/22). Трем пациентам проведено хирургическое лечение по поводу катаракты в возрасте 11, 15 и 16 лет. Частота катаракты не зависела от нозологической формы гипокальциемии (табл. 1).

Анализ длительности заболевания у пациентов с катарактой и без неё показал наличие статистически значимых различий только по предполагаемой длительности заболевания (p=0,018) (табл. 8). Также катаракта была ассоциирована с нефрокальцинозом (табл. 4).

**Table:** Таблица 8. Сравнительная характеристика признаков пациентов с наличием и отсутствием катаракты

Признак	Группа с катарактой	Группа без катаракты	P, U-test
N	Me [ Q1; Q3]	N	Me [ Q1; Q3]
Длительность заболевания с момента впервые задокументированной гипокальциемии, лет	24	4,8 [ 1,9; 8,2]	105	3,6 [ 1,3; 7,5]	0,356
Предполагаемая длительность заболевания, лет	24	6,2 [ 4,6; 10,9]	105	4,3 [ 1,7; 8,2]	0,018

## ОБСУЖДЕНИЕ

## Патология почек

Одним из эффектов ПТГ на уровне почечных канальцев является снижение экскреции кальция. При дефиците ПТГ кальциурия возрастает, что может приводить к нефролитиазу и нефрокальцинозу. Терапия хронического ГПТ активными аналогами витамина D корректирует гипокальциемию, но не заменяет физиологические эффекты ПТГ и мало влияет на кальциурию, особенно у определенных групп пациентов, например, при патологии кальций-чувствительного рецептора [15][16]. Данные о частоте гиперкальциурии у детей при ГПТ ограничены, также затруднено сравнение с исследованиями взрослых пациентов из-за различий в методике: оценка суточной мочи у взрослых пациентов, разовой порции мочи у детей.

В нашем исследовании гиперкальциурия при достижении оптимальных значений кальция крови сохранялась у 60% (30/50) пациентов. Это наводит на мысль о пересмотре целевых уровней кальция, однако их снижение может усилить гиперфосфатемию, что может тоже служить причиной других осложнений. Все это может говорить о неоптимальности классической терапии гипопаратиреоза, необходимости внедрения в терапию новых препаратов, например, препаратов паратгормона или пересмотра схем терапии аналогами витамина D, более расширенного применения препаратов, снижающих уровень кальция в моче [17–19]. Ранее коллегами в ФГБУ «НМИЦ эндокринологии им. академика И.И. Дедова» была показана целесообразность суточного мониторинга кальция крови у пациентов с ГПТ для более оптимального подбора терапии в связи с выраженными суточными колебаниями кальция крови [20]. Колебания кальция крови могут вносить вклад в гиперкальциурию и развитие осложнений со стороны почек, в связи с чем апробация в будущем метода суточного мониторинга кальция у детей может быть полезной.

По данным Meola A. и соавт., гиперкальциурия выявлялась у 54,9% взрослых пациентов с ГПТ, коррелировала с уровнем кальция крови (р=0,044), но не ассоциировалась с длительностью ГПТ, функцией почек, концентрацией витамина D или наличием нефролитиаза [21]. Аналогично, нами не выявлено достоверной связи с уровнем креатинина, рСКФ, длительностью терапии и суточными дозами препаратов кальция и активных аналогов витамина D, но отмечалась тенденция к более высоким суточным дозам альфакальцидола у пациентов с гиперкальциурией: 2 мкг против 1,25 мкг у пациентов без гиперкальциурии.

Нами получено, что самая высокая частота гиперкальциурии определяется у пациентов с АДГ 1‑го типа и АПС 1‑го типа и не встречается вовсе у пациентов с ПГПТ. В случае с пациентами с АДГ 1‑го типа и ПГПТ полученные результаты объяснимы с точки зрения патогенеза этих заболеваний. В случае с пациентами с АПС 1‑го типа высокая частота гиперкальциурии не ясна. Можно предположить, что она связана с менее контролируемым течением ГПТ с развитием частых декомпенсаций, а также с возможной вариабельностью уровня кальция в течение дня, что может быть связано с нарушением всасывания на фоне аутоиммунной энтеропатии и влиянием других компонентов заболевания и их терапии на всасывание аналогов витамина D и кальция.

В нашей когорте двусторонний нефрокальциноз выявлен у 58% (55/95) пациентов с ГПТ и у 22% (7/32) пациентов с ПГПТ. В исследовании Mazoni L. кальциноз почек выявлен у 29,2% взрослых пациентов с послеоперационным ГПТ, при этом корреляции с гиперкальциурией не выявлено, а рСКФ не отличалась от контрольной группы [22]. Наши данные также не подтвердили наличие связи между гиперкальциурией и нефрокальцинозом, что предполагает наличие дополнительных механизмов, участвующих в формировании нефрокальциноза, например, хроническая гиперфосфатемия. Однако нами выявлена связь нефрокальциноза с длительностью заболевания и терапией активными формами витамина D и препаратами кальция.

По данным двух независимых когортных исследований, риск бессимптомного и симптоматического нефролитиаза у пациентов с послеоперационным ГПТ увеличивается примерно в восемь и четыре раза соответственно [21][23]. В нашем исследовании мочекаменная болезнь диагностирована в 3,9% случаев и только у пациентов с нехирургическими формами ГПТ, при ПГПТ она не встречалась. Симптоматическое течение МКБ зарегистрировано в одном случае и потребовало проведения литотрипсии. Результаты нашего исследования свидетельствуют о формировании осложнений со стороны почек уже в детском возрасте и указывают на высокую распространенность бессимптомных изменений, что подчеркивает необходимость регулярного инструментального скрининга для ранней диагностики и профилактики осложнений.

В исследовании у пациентов с нехирургическим ГПТ риск возникновения нефрокальциноза и нефролитиаза не отличался от контрольной группы, тогда как риск почечной недостаточности у пациентов был увеличен в шесть раз [4]. В нашем исследовании не выявлено статистически значимого снижения функции почек у пациентов с нефрокальцинозом. Результаты когортного исследования Swartling O. свидетельствуют о значительном повышении риска развития ХБП, мочекаменной болезни и госпитализаций у пациентов с хроническим ГПТ [16]. В российской взрослой когорте пациентов с ГПТ нефрокальциноз был диагностирован у 10,7% пациентов и чаще носил двусторонний характер (93,5%), а у 17,4% пациентов наблюдалось значимое снижение рСКФ, соответствующее ХБП 3а-5 стадия [24]. Отсутствие связи между нефрокальцинозом и снижением функции почек в нашей когорте может быть связано с детским возрастом и относительно коротким периодом наблюдения.

## Синдром Фара

Синдром Фара представляет собой патологическую кальцификацию базальных ганглиев и других структур головного мозга, возникающую вторично на фоне нарушений минерального обмена [4].

В исследовании Zavatta G. кальцификация, по данным КТ, выявлялась у 25,4% пациентов с хроническим ГПТ, при этом при нехирургических формах встречалась в 5,1 раза чаще и ассоциировалась с низким уровнем кальция и сниженным кальций-фосфорным соотношением в сыворотке крови [25]. Goswami и соавт. сообщили о кальцификация у 73,8% пациентов при идиопатическом ГПТ, которая также ассоциировалась с низким кальций-фосфорным соотношением. Чаще всего минерализация отмечалась в базальных ганглиях, далее по частоте — граница серого и белого вещества, паренхима мозжечка, таламус и зубчатые ядра [26]. В нашем исследовании кальцификация структур головного мозга была сопоставимой — 76% (53/70), чаще с локализацией в базальных ганглиях, лобных и теменных долях, а также в мозжечке.

Wang C. с соавт. в 2012 г. впервые идентифицировали дефект гена SLC20A2, кодирующего натрий-фосфатный котранспортер III типа (PIT2), у 7 пациентов с идиопатической семейной кальцификацией головного мозга. Нарушение функции данного гена приводит к отложению фосфатов кальция в базальных ганглиях и, по мнению авторов, гиперфосфатемия может играть ключевую роль в формировании внескелетных кальцификаций при ГПТ [27]. В нашей когорте у пациентов с синдромом Фара отмечено статистически значимое повышение соотношения уровня фосфата к верхней границе нормы и тенденция к более высоким абсолютным значениям фосфата, что подтверждает роль нарушений фосфорного обмена. Вместе с тем низкая частота интракраниальных кальцификаций (9,6%) у пациентов с длительной гиперфосфатемией при хронической болезни почек в исследовании Savazzi G. наводит на мысль о многофакторном генезе синдрома Фара в случае ГПТ [28].

По данным других авторов частота синдрома Фара зависит от формы заболевания: при аутосомно-доминантной гипокальциемии — 38%, при идиопатическом ГПТ — 52–72%, при псевдогипопаратиреозе — до 100% [6][26][29]. В рамках нашего исследования кальцификация структур головного мозга наблюдалась у 88% пациентов с АДГ 1‑го типа, 72% — с АПС 1‑го типа, 70% — с ПГПТ и у всех обследованных пациентов с идиопатическим ГПТ.

Клиническое значение кальцификаций до конца не изучено. Вопрос о наличии взаимосвязи объема и локализации обызвествлений с неврологическими и нейропсихиатрическими проявлениями остается спорным и малоизученным.

## Катаракта

Предполагается, что отложение фосфатных кристаллов кальция в хрусталике способствует формированию катаракты у пациентов с ГПТ/ПГПТ. В нашей когорте пациентов детского возраста с ГПТ/ПГПТ распространенность катаракты составила 18,6%, а в российской когорте взрослых пациентов с хроническим ГПТ — 34,7% [24]. В нашем исследовании получилось, что у пациентов с катарактой предполагаемая длительность заболевания выше, чем у пациентов без катаракты, но при этом нет различий по длительности заболевания по впервые зафиксированной гипокальциемии, что может говорить о том, что именно длительная гиперфосфатемия, которая бывает до начала терапии, является главной причиной развития катаракты, а не сама длительность заболевания. Это дополнительно подтверждается тем, что у 7 из 24 пациентов катаракта развилась уже к моменту диагностики заболевания, то есть вероятным фактором была длительная гиперфосфатемия. В крупном когортном исследовании значимых различий риска развития катаракты у пациентов с послеоперационным ГПТ в сравнении с контрольной группой здоровых людей не получено (р=0,52) [30]. А относительно пациентов с наследственными формами ГПТ и идиопатическим ГПТ по сравнению со здоровым контролем были получены другие данные: риск возникновения катаракты в 4 раза выше по сравнению с группой контроля [4]. Мы предполагаем, что такое различие между пациентами с послеоперационным ГПТ и наследственными формами ГПТ может быть связано с тем, что первых пациентов начинают лечить сразу после операции, и у них не возникает длительного периода значимой гиперфосфатемии. А вот у пациентов с наследственными формами из-за отложенной диагностики такое может быть. Также может иметь значение возраст манифестации заболевания и особенности хрусталика у детей.

Распространенность катаракты в работе Goswami R. среди пациентов с идиопатическим ГПТ составила 51%, и катаракта коррелировала с кальцификацией базальных ганглиев [26]. Этот вывод логичен в связи с тем, что причина развития этих состоянии, вероятнее всего, связана с гиперфосфатемией. В нашем исследовании достоверной связи между катарактой и синдромом Фара не получено, однако распространенность катаракты в группе пациентов с синдромом Фара была в четыре раза выше, чем в группе без него. Полученные данные указывают на тенденцию к более высокой частоте катаракты у пациентов с синдромом Фара, однако для подтверждения этой ассоциации необходима более крупная выборка. Но в нашей группе получена статистически достоверная связь между катарактой и нефрокальцинозом. Эти два состояния также могут иметь общий патогенез и быть связаны с гиперфосфатемией.

По данным других исследователей, катаракта, возникающая при ГПТ, чаще всего имеет заднесубкапсулярную локализацию [26][31]. В нашем исследовании у детей чаще поражался кортикальный слой, что может быть обусловлено особенностями хрусталика в детском возрасте. Но заднекапсулярная локализация среди наших пациентов тоже встречалась часто.

## Ограничения исследования

## ЗАКЛЮЧЕНИЕ

Хронические осложнения выявлены у большинства пациентов. Нефрокальциноз и гиперкальциурия достоверно чаще наблюдались при АПС 1‑го типа и АДГ 1‑го типа. Нефрокальциноз ассоциировался с длительностью заболевания и терапией активными метаболитами витамина D и препаратами кальция. Гиперкальциурия не являлась достоверным предиктором нефрокальциноза. Высокая экскреция кальция с мочой сохранялась у 60% детей даже при оптимальном уровне кальция в крови. Эти данные указывают на сложность контроля минерального обмена и необходимость дальнейшего изучения подходов к терапии ГПТ. Синдром Фара диагностировался наиболее часто, преимущественно с поражением базальных ганглиев, и был ассоциирован с гиперфосфатемией. Катаракта и нефролитиаз встречались реже, без значимых различий между нозологическими формами. Систематизация данных об осложнениях имеет клиническое значение для оптимизации ведения и наблюдения пациентов с ПГПТ и различными формами ГПТ.

## ДОПОЛНИТЕЛЬНАЯ ИНФОРМАЦИЯ

Источники финансирования. Государственное задание №123021300171-7 «Хронический послеоперационный и нехирургический гипопаратиреоз: предикторы осложнений заболевания, контроль диагностики, лечения и мониторинга пациентов с использованием систем поддержки принятия врачебных решений» (2023–2025 гг.).

Конфликт интересов. Авторы декларируют отсутствие конфликта интересов.

Участие авторов. Все авторы одобрили финальную версию статьи перед публикацией, выразили согласие нести ответственность за все аспекты работы, подразумевающую надлежащее изучение и решение вопросов, связанных с точностью или добросовестностью любой части работы.
